# ^1^H Nuclear Magnetic Resonance of Pig Seminal Plasma Reveals Intra-Ejaculate Variation in Metabolites

**DOI:** 10.3390/biom10060906

**Published:** 2020-06-15

**Authors:** Yentel Mateo-Otero, Pol Fernández-López, Sergi Gil-Caballero, Beatriz Fernandez-Fuertes, Sergi Bonet, Isabel Barranco, Marc Yeste

**Affiliations:** 1 Biotechnology of Animal and Human Reproduction (TechnoSperm), Institute of Food and Agricultural Technology, University of Girona, E-17003 Girona, Spain; yentel.mateo@udg.edu (Y.M.-O.); beatriz.fernandez@udg.edu (B.F.-F.); sergi.bonet@udg.edu (S.B.); 2 Unit of Cell Biology, Department of Biology, Faculty of Sciences, University of Girona, E-17003 Girona, Spain; 3 Theoretical and Computational Ecology Group, Centre for Advanced Studies of Blanes (CEAB), Consejo Superior de Investigaciones Científicas (CSIC), E-17300 Girona, Spain; pfernandez@ceab.csic.es; 4 NMR Facility, Research Technical Services (STR), University of Girona, E-17003 Girona, Spain; sergio.gil@udg.edu; 5 Department of Medicine and Animal Surgery, Faculty of Veterinary Medicine, University of Murcia, E-30100 Murcia, Spain

**Keywords:** nuclear magnetic resonance, seminal plasma, metabolites, pig, intra-ejaculate variability

## Abstract

In pigs, ejaculate is expelled in fractions, mainly the sperm-rich fraction (SRF) and the post-SRF (PSRF), which differ in both sperm content and origin. In addition, intra-ejaculate variability between fractions in terms of sperm reproductive characteristics has been previously reported, the highest sperm quality being observed in the first 10 mL of the SRF (SRF-P1). As seminal plasma (SP) composition has been purported to influence sperm physiology, the aim of this study was to profile pig SP metabolite composition and to find putative differences between the ejaculate portions (SRF-P1, the rest of SRF [SRF-P2], PSRF) and entire ejaculate (EE). To this end, ejaculates (*n* = 8, one per boar) were collected in fractions and SP was analyzed using ^1^H Nuclear Magnetic Resonance spectroscopy. We identified 19 metabolites present in all ejaculate portions and the EE, and reported correlations between the metabolites. Additionally, and for the first time in mammals, we found intra-ejaculate variability in the SP metabolites, observing different relative abundances in choline, glycerophosphocholine and glycine. Regarding their influence in sperm physiology, we hypothesize that these metabolites may explain the specific reproductive characteristics of each ejaculate portion. Finally, the reported SP metabolites could serve as a first steppingstone in the study of quality, functionality, and fertility biomarkers.

## 1. Introduction

To date, artificial insemination (AI) remains the most widely used reproductive biotechnology in the swine industry [1]. Although pig ejaculates used to produce AI-doses are selected on the basis of sperm quality parameters (such as motility, morphology and plasma membrane integrity), not all selected ejaculates respond equally to liquid storage, freezing and sex-sorting, nor are fertility outcomes even when collected from the same boar [2,3,4]. Because of this intra-boar variability, efforts have been made to uncover accurate sperm quality and fertility biomarkers that could help select the most suitable ejaculates for AI. In this regard, the study of pig seminal plasma (SP) components is gaining relevance. The SP is a complex fluid resulting from the mixture of secretions from the testis, epididymis and accessory sexual glands, that directly interacts with sperm, at the time of ejaculation, and the female reproductive tract, once it is deposited [5,6]. Mounting evidence indicates that not only does SP carry, protect and nourish sperm, but also modulates their capacitation, energy production and fertilizing ability [7,8]. Moreover, once in the female reproductive tract, SP is able to modulate the immune response, conditioning the success of fertilization and subsequent pregnancy [9,10]. Consequently, differences in the composition of SP have been reported to exert an impact on sperm quality, function and in vivo fertility outcomes in pigs [6,11,12,13].

As in other mammalian species, such as humans [14] and horses [15], the pig ejaculate is expelled in well-defined fractions, highlighting: (1) the first fraction or sperm-rich fraction (SRF), which contains 80–90% of all ejaculated sperm and whose volume ranges from 70 to 100 mL; (2) the second fraction or post-SRF (PSRF), which contains 10–20% of the ejaculated sperm and whose volume is approximately 150–200 mL [6,16,17,18]. It has been suggested that, whereas the SP of the SRF is mainly composed of epididymal and prostate secretions, with a minor contribution from seminal vesicles, that of PSRF is primarily made up of secretions from seminal vesicles, with a minor proportion from the prostate [6,16,17,18]. Apart from differing in their sperm concentration, these ejaculate-fractions exhibit differences in molecular composition, both from a quantitative and a qualitative point of view [6,11,12,16,19,20]. The SRF can be further divided into two portions based on sperm concentration and functionality: the first 10 mL of the SRF (SRF-P1), and the rest of the SRF (SRF-P2) [21,22]. Previous studies have shown that sperm contained in SRF-P1 exhibit the highest motility and viability rates [21], withstanding liquid-storage better [23], and have better cryotolerance [13,16,24,25] than those recovered from the SRF-P2 or PSRF, the latter having the poorest outcomes. Differences in SP composition between ejaculate fractions/portions suggest that some of these differences in sperm performance could be due to this fluid [12,13,25]. Indeed, Perez-Patiño et al. (2016) identified differential expression of SP proteins between ejaculate-portions, which could be linked to sperm reproductive performance [20].

In the last years, the putative impact that SP could exert on pig sperm has acquired greater relevance due to the changes in the ejaculate collection systems in AI-centers. The traditional gloved-hand collection method, which allows the separation and discarding of the PSRF from the SRF [20,26], is being substituted by semi-automatic systems that collect the entire ejaculate (EE), including the PSRF [27]. These systems allow for a more hygienic collection and lower cost-labor for AI-centers [27], but imply a larger proportion of SP being present in the AI-dose. Although there are no studies evaluating the influence of this procedure shift on reproductive performance, there is evidence that SP from the PSRF is less cryoprotective for sperm in comparison to SP from the SRF [25], and sperm samples diluted in 50% SP had a reduced sex sorting efficiency in comparison to sperm diluted in 10% or no SP [26]. In addition, it has been demonstrated that SP from the PSRF exhibits lower total antioxidant capacity, whose values have been related to in vivo fertility outcomes [12]. These findings suggest that SP from different ejaculate fractions, and, consequently, collection method, could have a direct impact on sperm performance. Thus, studies aimed to perform an in-depth characterization of SP composition beyond the proteome profile are of the utmost importance for the swine industry.

The large-scale study of cellular metabolic products (metabolomics) reflects the downstream events of gene expression [28]; for this reason, some authors consider it to describe the phenotype rather than proteomics or genomics, because changes in the metabolome are amplified relative to changes in the transcriptome or proteome [29,30]. These molecules have been demonstrated to play a key role in several biological processes, including gamete quality and function [31]. In this regard, in-depth metabolomic analyses of human SP have revealed that several metabolites are related to sperm quality parameters, such as concentration, morphology and motility [32,33,34]. The relationship between SP metabolites and sperm functionality have also been reported in humans, showing that the contribution of metabolites such as uric acid, tryptophan or taurine, maintains reactive oxidative species (ROS) at low levels in this specie [35]. In addition, the relationship between SP metabolites and fertility has been evidenced in both humans [32,33,36,37,38,39] and cattle [40,41], with several metabolites that can explain male infertility in humans [32,33,36,37,38,39] or can potentially serve as biomarkers of bull fertility [40,41,42]. To the best of our knowledge, however, there is no information regarding the metabolomic profile of pig SP.

Based on all these data, the specific objectives of the present work are: (1) to characterize the metabolite composition of pig SP, and (2) to identify differentially expressed metabolites in SP recovered from different portions of the ejaculate (SRF-P1, SRF-P2 and PSRF) and the EE. To this end, SP from Large-White and Pietrain boars was analyzed by ^1^H Nuclear Magnetic Resonance (^1^H RMN) spectroscopy, due to the reproducible and high resolution information that this technique provides regarding the metabolomic composition of biofluids [28,43].

## 2. Materials and Methods

### 2.1. Boars and Ejaculates

Samples were provided by an AI Center (AIM Ibérica; Topigs Norsvin Spain SLU) located in Calasparra (Spain) that fulfills the current Spanish and European legislation for both commercialization of boar semen and animal health and welfare. The registration number in Spain that certifies the compliance with that legislation is ES300130640127 (August, 2006) and he EU registration number is ES13RS04P (July, 2012).

Ejaculates were obtained from eight healthy and sexually mature boars (aged 18–36 months) of different breeds (Large-White [*n*= 5 boars] and Pietrain [*n*= 3 boars]) belonging to one AI-center (AIM Iberica, Calasparra, Spain). Boars were housed in individual pens in an environmentally controlled building (15–25 °C) with *ad libitum* access to water and fed with a commercial diet according to the nutritional requirements for an adult boar [44]. Boars underwent regular semen collection (twice per week) to produce commercial AI-doses.

Ejaculates (*n* = 8, one ejaculate per boar) were collected in three separate portions: the first 10 mL of the SRF (SRF-P1), the rest of the SRF (SRF-P2) and the PSRF, using the gloved-hand method. A proportionate volume, based on the recovery volume of each fraction, was thereafter mixed to mimic an EE. All of the EEs used fulfilled the standards of sperm quantity and quality thresholds for the preparation of semen doses for use in AI-programs, i.e., more than 200 × 10^6^ spermatozoa/mL, 70% motile spermatozoa and 75% morphologically normal cells [45]. To isolate the SP, semen samples from each of the three ejaculate-portions and the EE were processed as indicated below.

### 2.2. Processing and Storage of SP

Immediately after collection, the three ejaculate-portions and the EE were centrifuged twice at 1500 g for 10 min at room temperature (Rotofix 32A, Hettich Centrifuge UK, Newport Pagnell Buckinghamshire, UK). The supernatant was microscopically examined (Eclipse E400, Nikon, Melville, NY, USA) to verify that it was sperm-free. Then, SP samples were aliquoted, and stored in 3 mL-cryotubes at −80 °C (Ultra Low Freezer, Haier Inc., Qingdao, China) until metabolomic analysis.

### 2.3. ^1^H RMN Analysis

Seminal plasma samples (300 µL) were thawed on ice, vortexed, and centrifuged thrice (12,000 g for 30 min at 4 °C) to remove any remaining cell contents. A 250-µL aliquot of the supernatant was mixed with 350 µL phosphate-buffered saline (10% D2O; Merck KgaA, Darmstadt, Germany; pH 7.4), and transferred into a 5 mm Wilmad^®^ NMR tube (Merck KgaA) to generate ^1^H NMR spectra.

^1^H NMR spectra of SP samples were acquired through an Avance III HD Nanobay 400 MHz spectrometer (Bruker, Billerica, MA, USA), equipped with a 5-mm BBOF probe at 298 K. ^1^H NMR measurements were performed using the pulse sequence cpmgpr1d, which includes presaturation and T2 filter. Free induction decays were acquired with 64 K data points, a spectral width of 8012 Hz and a resolution of 0.24 Hz. Water signal was attenuated using a weak presaturation pulse, calibrated at 50 Hz. ^1^H NMR spectra was recorded with 128 scans, with an interscan delay of 2.5 s.

1D NMR data were processed with TopSpin software version 3.6 (Bruker), with a line broadening of 1 Hz and 128 K data points and calibrated to sodium trimethylsilylpropanesulfonate (DSS) peak at 0.0 ppm. The SP metabolites were determined using ^1^H-NMR, 2D ^1^H-^1^H TOCSY, ^1^H-^13^C HSQC spectra, the Human Metabolome Database (HMDB; https://hmdb.ca/) [46] and the Biological Magnetic Resonance Data Bank (BRMB; http://www.bmrb.wisc.edu/) [47]. The selected metabolite signals were binned and integrated.

### 2.4. ^1^H RMN Data Statistical Analysis

The selected bins’ data, based on the signal intensity and peak isolation in order to achieve enough signal-to noise and to avoid spectral overlapping, were imported into MetaboAnalyst software (https://www.metaboanalyst.ca/) in order to perform statistical analysis [48]. Data was normalized by DSS, pareto-scaled (mean-centered and divided by the square root of the standard deviation of each variable), and their distribution was confirmed to be normal and homoscedastic. One-way analysis of variance (ANOVA) followed by a post-hoc Fisher LSD test was performed in order to analyze the variance of each metabolite through all SP samples. Then, a Pearson correlation analysis was carried out to determine the relationship between SP metabolites. After these multivariate analyses, principal component analysis (PCA) was used to reduce the dimensionality of data and assess which SP metabolites best explained the variance between ejaculate portions and the EE. To explore the effect of each metabolite, and because PC1 to PC4 explained 99% of the variability in the sample, the weighted sum of each metabolite loading value for PC 1 to PC4 was represented. Finally, the relative levels of metabolites in each portion of the ejaculate were also plotted. These last representations help classify the principal components and evaluate their impact over the different ejaculate-portions and the overall EE.

## 3. Results

### 3.1. Metabolomic Composition of Pig SP

**Figure 1:**
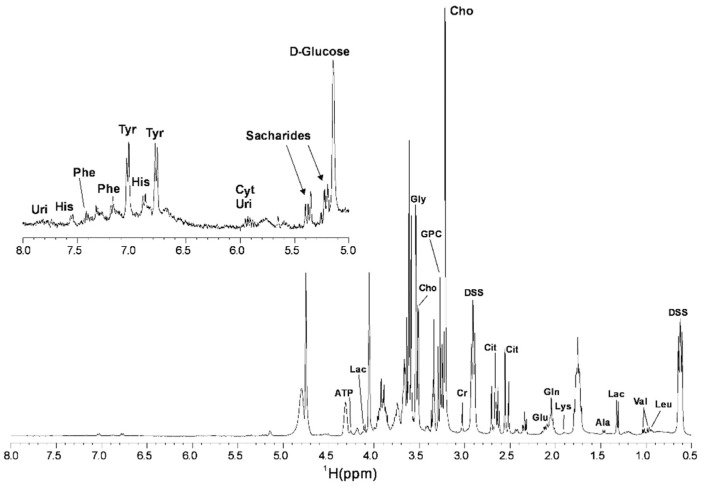
^1^H Nuclear Magnetic Resonance (^1^H NMR) Carr-Purcell-Meiboom-Gill (CPMG) spectrum of pig seminal plasma in phosphate buffer recorded at 400 MHz and 298 K. Sodium trimethylsilylpropanesulfonate (DSS) was used as internal standard. Uridine (Uri); Histidine (His); Phenylalanine (Phe); Tyrosine (Tyr); Citrate (Cit); Cytidine (Cyt); Adenosine Triphosphate (ATP); Lactate (Lac); Glycine (Gly); Choline (Cho); Glycerophosphocholine (GPC); Creatinine (Cr); Glutamic acid (Glu); Glutamine (Gln); Lysine (Lys); Alanine (Ala); Valine (Val); Leucine (Leu).

The ^1^H NMR spectrum (comprising between 0 and 8 ppm) obtained from pig SP samples is shown in Figure 1. A total of 24 identifiable spectral peaks were integrated and 19 metabolites were identified in all samples analyzed (Figure S1). Table 1 provides the resonance assignments of each metabolite identified. The metabolites were categorized in several chemical classes: amino acids (*n* = 10; Alanine [Ala], Glutamic acid [Glu], Glutamine [Gln], Glycine [Gly], Histidine [His], Lysine [Lys], Phenylalanine [Phe], Tyrosine [Tyr], Valine [Val] and Leucine [Leu]); salts (*n* = 2; Citrate [Cit] and Lactate [Lac]); nucleosides (*n* = 2; Uridine [Uri] and Cytidine [Cyt]); other organic compounds (*n* = 5; Adenosine Triphosphate [ATP], Choline [Cho], Creatinine [Cr], D-Glucose and Glycerophosphocholine [GPC]).

### 3.2. Comparison of SP Metabolites between Ejaculate-Portions and the EE

**Figure 2:**
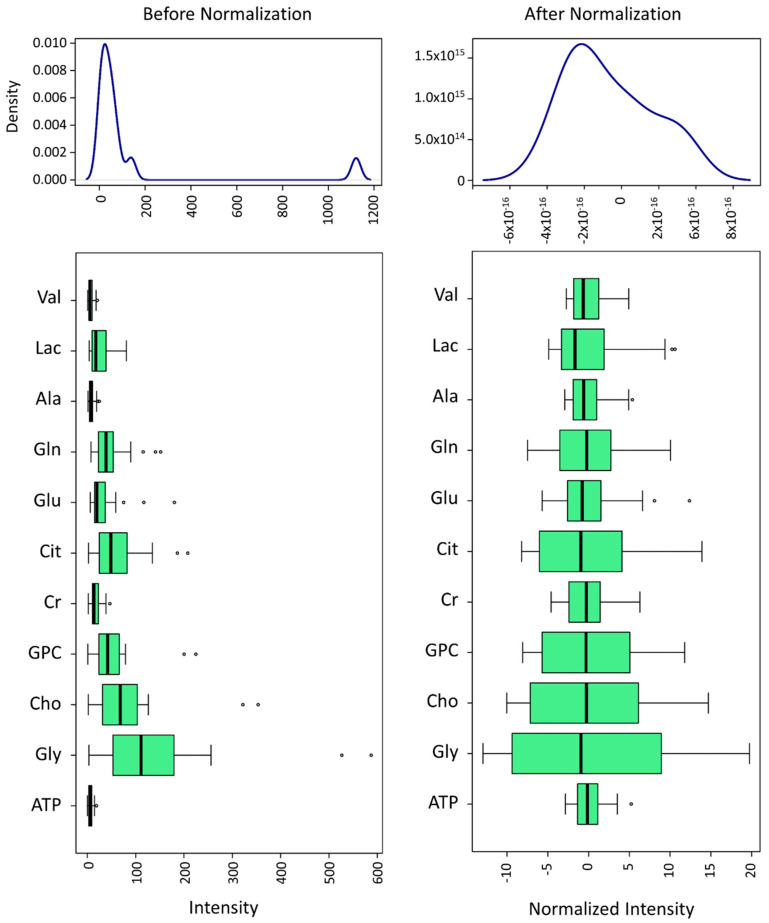
Selected metabolites’ spectral bin data normalization to DSS of pig seminal plasma, generated using MetaboAnalyst. Adenosine Triphosphate (ATP), Alanine (Ala), Choline (Cho), Citrate (Cit), Creatinine (Cr), Glutamic acid (Glu), Glutamine (Gln), Glycine (Gly), Glycerophosphocholine (GPC), Lactate (Lac) and Valine (Val). Normalization was performed with pareto-scaling and is shown on the left side. Empty dots in box-plots represent the outlier values both before and after normalization. Values were classified as outliers when their value was lower than 1.5 times the 25th percentile or higher than 1.5 times the 75th percentile.

To quantitatively compare the SP metabolites of each ejaculate-portion and the EE, 11 of the 24 identified spectral peaks (which corresponded to 11 different metabolites) were included in the statistical analysis. This selection was based on the signal intensity and peak isolation in order to achieve enough signal-to noise and to avoid spectral overlapping (metabolites indicated with an asterisk in Table 1). Then, data were normalized to DSS and pareto-scaled (Figure 2) for further analysis with MetaboAnalyst.

**Figure 3:**
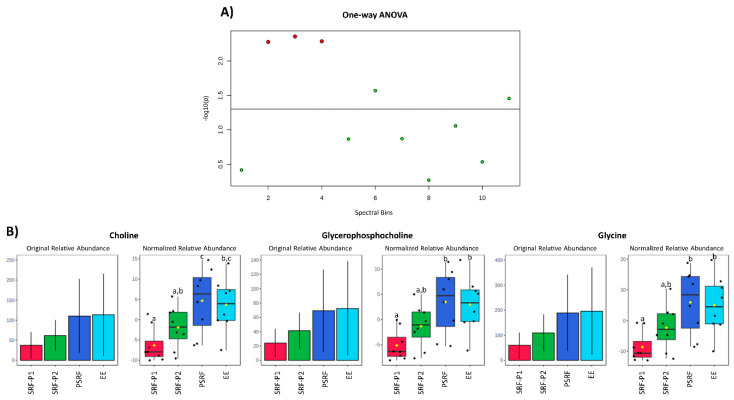
Comparison of the relative areas under each metabolite peak from the pig seminal plasma (SP) of different ejaculate-portions. (**A**) Graphical representation of one-way ANOVA, generated using MetaboAnalyst [48]. Red points represent metabolites whose relative values differ (*p* < 0.05) between at least two ejaculate-portions. Green points represent metabolites whose relative values do not differ (*p* > 0.05) between at least two ejaculate-portions. (**B**) Box plot representations of the original and the normalized relative abundance for the metabolites presenting differences between ejaculate-portions (first 10 mL from the sperm-rich fraction [SRF-P1], the rest of the sperm rich fraction [SRF-P2], the post-sperm-rich fraction [PSRF] and the entire ejaculate [EE]). Different superscripts (a–c) indicate differences (*p* < 0.05) between ejaculate-portions.

**Figure 4:**
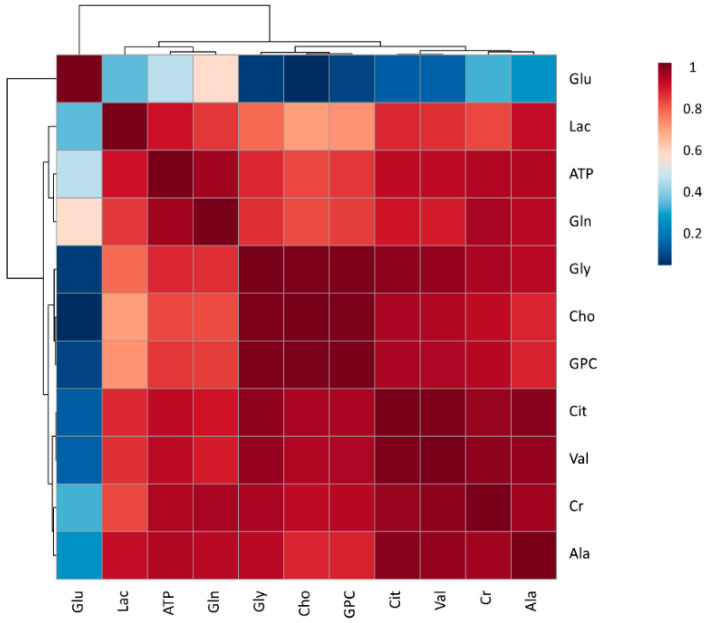
Correlation plot of the eleven analyzed metabolites identified in boar seminal plasma, generated using MetaboAnalyst. The color saturation of red to blue represents the Pearson correlation coefficients (r) between metabolites, from 0 to 1, respectively. Adenosine Triphosphate (ATP), Alanine (Ala), Choline (Cho), Citrate (Cit), Creatinine (Cr), Glutamic acid (Glu), Glutamine (Gln), Glycine (Gly), Glycerophosphocholine (GPC), Lactate (Lac) and Valine (Val).

First, the relative areas under each peak were compared between ejaculate-portions and the EE. The relative abundance of three metabolites (Gly, Cho and GPC) differed (*p* < 0.01) between ejaculate-portions and the EE (Figure 3A). In general, these three metabolites exhibited a similar pattern, showing lower relative abundance in SP from the SRF, compared to the PSRF and the EE (Figure 3B). Specifically, the relative levels of Gly and GPC were lower (*p* < 0.01) in SP from the SRF-P1 (−8.6 ± 5.01 and −5.1 ± 3.11, respectively) than in SP from the PSRF (5.9 ± 10.61 and 3.5 ± 6.39, respectively) and the EE (5.0 ± 9.47 and 3.0 ± 5.49, respectively). Similarly, the relative levels of Cho were lower (*p* < 0.01) in SP from the SRF-P1 (−6.3 ± 4.29) and SRF-P2 (−1.9 ± 5.22) than in SP from the PSRF (4.7 ± 8.03). Moreover, SP from the SRF-P1 (−6.3 ± 4.29) also showed lower Cho levels (*p* < 0.01) compared to SP from the EE (3.6 ± 6.62). In addition, regardless of the portion of the ejaculate analyzed, our results revealed a strong positive correlation between these three metabolites (Pearson’s coefficient’s [r] higher than 0.99; Figure 4). In general, the metabolites identified exhibited a very strong to moderate positive correlation (r values between 0.60 and 0.99), except for Glu, in which a weak positive correlation was found (r < 0.5; Figure 4).

**Figure 5:**
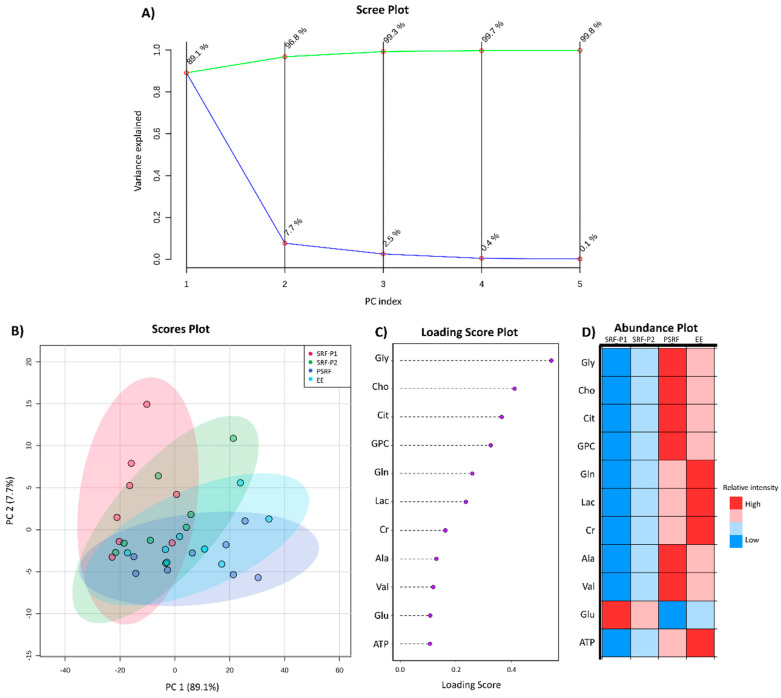
Intra-ejaculate analysis of the variance of pig seminal plasma. (**A**) Scree plot showing the individual variance contribution for each principal component (PC) in blue and the cumulative variance explained along the PCs in green. (**B**) PCA scores plot showing the distribution of the samples in PC1 and PC2. The colored areas represent the 95% confidence interval, depicting which ejaculate-portion each sample belonged to (the first 10 mL from the sperm-rich fraction [SRF-P1], the rest of sperm rich fraction [SRF-P2], the post-sperm-rich fraction [PSRF] and the entire ejaculate [EE]). (**C**) Representation of the weighted sum of PCA loadings from the first three PCs. (**D**) Abundance plot representing the relative levels of each metabolite for every ejaculate portion. Color variation from red to blue correspond to high and low relative levels, respectively. Adenosine Triphosphate (ATP), Alanine (Ala), Choline (Cho), Citrate (Cit), Creatinine (Cr), Glutamic acid (Glu), Glutamine (Gln), Glycine (Gly), Glycerophosphocholine (GPC), Lactate (Lac) and Valine (Val).

To analyze the distribution of the sample set, a PCA was carried out. The scree plot revealed that the cumulative principal component (PC) score of two PC explained 96.8% of the data variance, achieving more than 99% in the first three PCs (Figure 5A). The PCA score plot of PC1 (89.1%) and PC2 (7.7%) evidenced four different groups, corresponding to the three different portions of the ejaculate and the EE (Figure 5B). While the SRF-P1 was mostly explained by PC2, the PSRF and EE were mostly determined by PC1. The SRF-P2 was found between these two regions. The weighted sum of the loadings from PC1–3 was represented as a loading score in order to evaluate the implication of each metabolite in the variance of the SP sample. The same metabolites detected in the ANOVA analysis to differ between the samples (Cho, Gly and GPC), together with Cit, exhibited the highest loading scores (0.54 for Gly, 0.41 for Cho, 0.36 for Cit and 0.32 for GPC; Figure 5C), indicating that these metabolites contribute the most to the sample variance.

Finally, to characterize the contribution of each metabolite to the different ejaculate fractions, an abundance plot was created. The abundance plot showed a pattern similar to the PCA distribution, with Gly, Cho and GPC exhibiting the highest abundance in the PSRF and the lowest in SRF-P1 (Figure 5D).

## 4. Discussion

Traditionally thought to be a mere vehicle for sperm deposition in the female reproductive tract, studies over the years have shown SP to modulate sperm function, regulate the female reproductive environment, and even affect offspring health [6,49,50,51]. In-depth studies of SP composition, such as the one carried out here, can aid in our understanding of the molecular mechanisms behind these effects. To the best of our knowledge, this is the first study that has characterized the metabolites of pig SP, which comprises a total of 19 compounds. Perhaps more importantly, the data show an intra-ejaculate variability in the metabolites as well as a correlation between each other.

The assessment of male fertility has traditionally focused on the evaluation of sperm attributes, such as motility, morphology and viability [6,52]. However, these sperm quality parameters possess limited accuracy when selecting pig ejaculates for AI, since differences in the ability to withstand preservation and fertility outcomes still remain remarkable between ejaculates from the same male [53]. Thus, novel biomarkers of ejaculate quality are needed. In this regard, the study of SP components is gaining interest. In both men [32,33,36,37,38,39] and bulls [40,41,42], SP metabolites have been linked to sperm function, pointing at a possible role as markers of sperm quality and/or fertility. These studies evidence that an in-depth metabolomic profile of SP could provide a valuable list of potential sperm quality and fertility biomarkers for AI-boars. Among the analytical methods for metabolite profiling, ^1^H NMR spectroscopy is emerging as one of the most used, because it provides a uniform metabolite detection of equal sensibility in mixture samples, such as SP [28]. Therefore, the present study performed a characterization of the metabolite composition of pig SP using ^1^H NMR spectroscopy.

Our data shows that the pig SP is comprised by 19 metabolites, which include amino acids, salts, nucleosides, and other organic compounds. The ^1^H NMR spectra obtained for pig SP is similar to that of human SP, with all identified metabolites in pig SP also present in human SP [33,38,39]. In some of these human studies, a higher number of spectral peaks (between 31–33 peaks) and metabolites were identified compared with the results reported here, which could be due to differences in the preparation of SP samples, or in the ^1^H NMR spectrometer used [33,38]. The similarity between pig and human SP metabolite composition was surprising considering their different proteomic profiles [7,20]. These metabolic similarities, together with the fact that both species expel the ejaculate in defined fractions, could make the pig a good model for humans in this regard. On the other hand, no similarities were found between the ^1^H NMR spectra of bovine [40] and porcine SP, species that also differ in the protein composition of their SP [7,54].

To the best of our knowledge, this is also the first work that has evaluated the potential relationship between SP metabolites. In general, our results revealed that they were positively correlated. This correlation was strong between Gly, Cho and GPC, and weak between all SP metabolites and Glu. Correlations between metabolites result from the reactions and regulation of networks, and strong positive correlations may indicate a rapid equilibrium, enzyme dominance or similar regulation [55,56]. Thus, if amino acids share substrates or have a tight regulation, they are usually positively correlated [56]. Because Ala, Val, Gly, Gln and Glu are glucogenic metabolites involved in the Krebs cycle, their positive correlation was expected [57,58]. However, Glu was found to have a weak correlation with all other metabolites present in SP. Because Glu is involved in many pathways, such as neutroansmission, inflammation or ammonia assimilation [58], its activation or inhibition may be responsible for the weak correlation observed. Moreover, Cit is an intermediate product of the Krebs cycle, an energy pathway in which ATP is the final product [58]. Thus, the role of both Cit and ATP in such pathways may explain the positive correlation observed between them and with most of the analysed amino acids. On the other hand, the strong correlation between Gly, Cho and GPC could be explained considering that Cho can synthesize both Gly (using sarcosine as an intermediate product) [58] and GPC [59]. Finally, Gly has also been reported to produce Cr, thus the observed positive correlation was expected [58]. All this evidence suggests that the positive relationship observed between all of our identified metabolites is due to all of them being implicated in energy productions and lipid biosynthesis (which at the same time are linked pathways). On the other hand, because most of the metabolites showed a strong to moderate correlation between them, the assessment of a reduced number of SP metabolites as fertility biomarkers could be considered instead of having to perform a whole metabolomic study.

Previous studies suggest that the epididymis mainly contributes to the SP of the SRF-P1, the prostate to the SP of the SRF-P2, and the seminal vesicles to the SP of the PSRF [6,16,17,18]. While our results revealed that all SP metabolites are present in all portions of the ejaculate, suggesting that the epididymis and all of the accessory sexual glands are able to secrete all of the identified metabolites, their relative quantities differed. The PCA analysis showed four distribution patterns which correspond to the different portions of the ejaculate and the EE. The intensity plot not only confirmed the aforementioned differences, but also identified which portion contributed the most to the overall EE for each metabolite. Among the 11 metabolites analyzed, Gly, Cho and GPC showed different relative levels between separate ejaculate portions. The PSRF presented the highest relative levels of these metabolites, while they were lowest in the SP from the SRF-P1. Considering that the SP from each portion is mainly composed of the secretions of specific accessory sex glands or the epididymis [22], the seminal vesicles are probably the main secretors of Gly, Cho and GPC, since the SP of the PSRF comes mainly from these glands. On the contrary, the epididymis, which mainly contributes to the SP of the SRF-P1 [13,16,22], may barely partake in the secretion of these three SP metabolites. These results would be in agreement with those reported in humans by Lynch et al. [60], in which the ^1^H NMR spectrum of prostatic and seminal vesicle fluids surgically obtained also revealed a higher concentration of GPC, and also lactate, in the seminal vesicle fluid compared to the prostatic fluid. All of the presented data, together with the fact that seminal vesicles represent 80–90% of the total SP protein content [61], would suggest that seminal vesicles could be the main contributors to the secretion of these SP metabolites.

In pigs, sperm from different ejaculate portions have been shown to differ in their motility and viability [21], as well as in their ability to withstand storage procedures [13,16,23,24,25]. In general, sperm contained in the SPF-P1 have better quality than those recovered from other fractions and, based on in vitro studies, they have been suggested to form the bulk of the oviductal sperm reservoir [21]. In humans, the SRF has also been reported to contain sperm with better motility and viability [62,63,64], and lower DNA fragmentation [14,15]. The specific composition of the SP from each ejaculate fraction/portion has been proposed to explain the difference in sperm performance in humans [65] and in pigs [6,12,13]. In this regard, a previous study demonstrated that while SP from the SRF possesses a cryoprotective effect on pig sperm, that from the PSRF has a negative impact and leads to increased endogenous H_2_O_2_ levels [25]. In effect, SP from different portions of the ejaculate have different antioxidant compositions, which has been linked to the differences observed in sperm cryotolerance [13]. Moreover, high total antioxidant capacity has been positively correlated to higher fertility outcomes, probably because it improves the sperm fertilizing ability [12]. Differences in the proteomic composition of SP between ejaculate portions has also been reported, uncovering that certain proteins linked to sperm reproductive performance are differently expressed between ejaculate portions [20]. Interestingly, the present study found a well-defined spatial distribution in PCA for each portion, in which the main metabolites contributing to the sample variability were Gly, Cho, Cit and GPC. The fact that PCA distribution has arisen in a completely unsupervised and statistically significant manner, together with the finding that the portions’ confidence intervals are not completely overlapped, adds robustness to the idea that different ejaculate portions present a specific metabolite composition. Additionally, differences in SP metabolites between ejaculate portions were also evidenced, particularly in the relative abundance of Cho, GPC and Gly. Therefore, we hypothesize that these differences could contribute to explaining the variability existing between ejaculate portions in terms of sperm physiology. In fact, these SP metabolites have been reported to contribute to physiological processes that require very large amounts of energy production [66,67,68].

The amino acid Gly has been related to the initiation of the acrosome reaction (AR) in pig, mouse, hamster and human capacitated sperm [66,69,70], probably via mechanisms involving the sperm Gly receptor [66,71]. Although appropriate concentrations of Gly are probably required for AR, excessive levels could have detrimental effects for sperm. Incubation of sperm from the SRF-P1, with SP from the SRF-P2 and PSRF, has a detrimental effect on the percentage of sperm with intact plasma membranes [16]. Considering that, in this study, the PSRF exhibited the highest relative abundance of Gly, this metabolite could be driving, in part, the negative effect on plasma membrane integrity observed by Saravia et al. [16]. The premature capacitation and acrosome exocytosis resulting from the sperm membrane destabilization may translate into lower fertility rates from samples that include SP from the PSRF. Indeed, abnormal amounts of Gly in SP have been linked to unexplained male infertility [36].

On the other hand, both GPC and Cho are metabolites involved in the metabolism of glycerophospholipids. Both of these metabolites are involved in two key aspects of sperm physiology: capacitation and motility. In rats, hydrolysis of GPC by phospholipase C has been shown to occur during capacitation and AR, this lipid being one of those that decrease the most after the onset of capacitation [67]. In addition, the accumulation of lipid metabolites occurring during the AR has been proposed to have an implication in sperm–oocyte interaction and gamete fusion [67]. Regarding motility, a negative correlation was observed between sperm motility and GPC abundance in men SP [72]. In agreement with this data, in our study, GPC’s relative quantity was highest in the PSRF, which contains sperm with lower motility than the SRF [21]. However, the oxidation of Cho to form betaine via Choline Dehydrogenase produces electron transport chain substrates, which ultimately participate in ATP production, allowing processes such as sperm motility [68]. In this regard, Cho has been described as an energy source for sperm motility in *Drosophila Melanogaster* [73]. Thus, it is not clear why, in our study, a lower concentration of this metabolite was found in SP from the SRF than the PSRF. One explanation could come from the production of ROS. Since both motility and AR are oxidative processes, large amounts of Cho and GPC could result in the excessive production of ROS [74,75]. Although appropriate levels of ROS are required for sperm hyperactivation, AR or gamete fusion [76], excessive ROS may cause motility reduction, lipid peroxidation, DNA damage and apoptosis-like events [77,78]. In this context, it is worth mentioning that SP from the PSRF has been shown to be rich in ROS [15] and that the highest levels of H_2_O_2_ (the main ROS generated by pig spermatozoa) and lipid peroxidation have been found in sperm from the PSRF [13]. These results could contribute towards explaining the poor quality of sperm contained in this fraction, and suggest that the inclusion of the PSRF in AI-doses might have detrimental effects for the overall sperm population. The relative amount of GPC and Cho is typically reported in experimental and clinical NMR human studies because it tends to be altered by pathological conditions [79,80]. Some studies have proposed the GPC/choline ratio as a way of discriminating between different forms of spermatogenic failure, infertility and male reproductive tract [60,80]. Moreover, other studies on humans propose GPC as a biomarker for infertility [79], since abnormal GPC levels have been related to male infertility [37,38,79,81]. These studies suggest that Cho and GPC could be potential fertility biomarkers for AI-boars.

To sum up, GPC, Cho and Gly seem to contribute to the regulation of sperm physiology in processes such as motility, capacitation and AR. As aforementioned, SP from PSRF has been extensively suggested to have a negative impact upon sperm reproductive characteristics. Since different relative metabolite abundances between portions were observed, the highest levels for these three metabolites being found in the PSRF, we suggest that GPC, Cho and Gly may contribute towards explaining the sperm function variability between ejaculate portions. What is more, the three metabolites have previously been correlated with different forms of male infertility and suggested as putative fertility biomarkers. Therefore, further studies should elucidate whether GPC, Cho and Gly could also be used as SP biomarkers for pig sperm quality and fertilizing ability.

## 5. Conclusions

In conclusion, the present study describes the metabolomic composition of pig SP, showing intra-ejaculate variability. The variability in the metabolite abundances of SP between the different portions of the ejaculate suggests that certain metabolites, such as Cho, GPC and Gly, could be linked to sperm fertilizing ability. Due to the processes in which these metabolites are involved, together with previous evidence on specific sperm performance for each portion, SP from PSRF may have a detrimental effect on sperm functionality. On the other hand, this study provides a list of metabolites of pig SP which could serve as a first steppingstone for further studies to uncover the potential of SP metabolites as biomarkers of sperm quality, functionality and fertility in AI-boars. Moreover, because of the high correlations between SP metabolites, it would be possible to assess a reduced number of metabolites as biomarkers of fertility or sperm quality biomarkers instead of performing an in-depth metabolomic analysis of the samples. Progress in this field will likely contribute, not only to the improvement of artificial reproductive technologies in the swine industry, but also to the understanding of sperm physiology. Moreover, based on the similar behavior patterns of human sperm performance between ejaculate fractions and the similar metabolite composition of the SP, the pig could be an appropriate model for the study of the role of SP metabolites in reproductive physiology.

## Figures and Tables

**Table 1:** ^1^H NMR spectrum assignments of pig seminal plasma metabolites. Metabolites used in the statistical analysis are indicated by an asterisk (*).

Metabolite	Chemical Shift (ppm)
Uridine	7.83
Histidine	7.55
Phenylalanine	7.41
Phenylalanine	7.18
Tyrosine	7.04
Histidine	6.89
Tyrosine	6.77
Cytidine	5.93
Uridine	5.84
D-Glucose	5.14
ATP *	4.25
Lactate	4.10
Glycine *	3.54
Choline *	3.51
GPC *	3.27
Choline	3.22
Creatinine *	3.03
Citrate *	2.53
Glutamic acid *	2.11
Glutamine *	2.05
Lysine	1.91
Alanine *	1.47
Lactate *	1.32
Valine *	1.03
Valine/Leucine	0.98
Leucine	0.95

## Supplementary Materials

The following are available online at https://www.mdpi.com/2218-273X/10/6/906/s1, Figure S1: ^1^H NMR spectra of seminal plasma obtained from different portions of the pig ejaculate (first 10 mL from the sperm-rich fraction [SRF-P1; A], the rest of sperm rich fraction [SRF-P2; B], the post-sperm-rich fraction [PSRF; C]) and the entire ejaculate (D) recorded at 400 MHz and 298K.
