# Supplementary material for: 1H Nuclear Magnetic Resonance of Pig Seminal Plasma Reveals Intra-Ejaculate Variation in Metabolites
Source: Biomolecules. 2020 Jun 15;10(6):906. doi: 10.3390/biom10060906 (PMC7355445; doi:10.3390/biom10060906)
Supplement: Supplementary file 1 [file biomolecules-10-00906-s001.pdf]

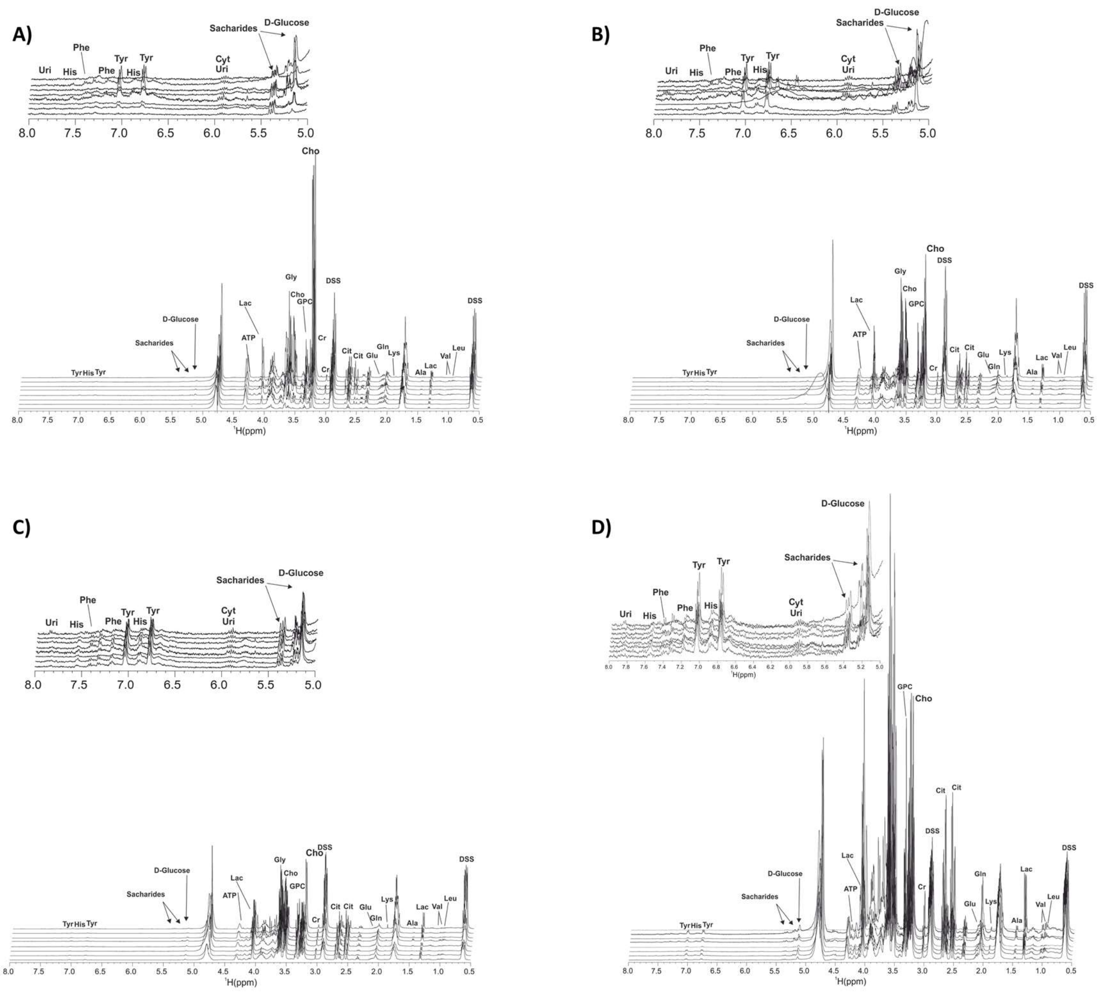

Figure S1: ^{1}H NMR spectra of seminal plasma obtained from different portions of the pig ejaculate (first 10 ml from the sperm-rich fraction [SRF-P1; **A**], the rest of sperm rich fraction [SRF-P2; **B**], the post-sperm-rich fraction [PSRF; **C**]) and the entire ejaculate (**D**) recorded at 400 MHz and 298K.
